# The functions and prognostic value of Krüppel‐like factors in breast cancer

**DOI:** 10.1186/s12935-022-02449-6

**Published:** 2022-01-15

**Authors:** Ke-Yun Zhu, Yao Tian, Ying-Xi Li, Qing-Xiang Meng, Jie Ge, Xu-Chen Cao, Ti Zhang, Yue Yu

**Affiliations:** 1 Department of Hepatobiliary Surgery, Liver Cancer Research Center, Tianjin Medical University Cancer Institute and Hospital, National Clinical Research Center for Cancer, Tianjin, 300060 China; 2 Key Laboratory of Cancer Prevention and Therapy, Tianjin’s Clinical Research Center for Cancer, Tianjin, 300060 China; 3 Department of General Surgery, Tianjin Medical University General Hospital, Tianjin, 300052 China; 4 Department of Pathogen Biology, School of Basic Medical Sciences, Tianjin Medical University, Tianjin, 300070 China; 5 Department of Radiobiology, Tianjin Medical University Cancer Institute and Hospital, National Clinical Research Center for Cancer, Tianjin, 300060 China; 6 The First Department of Breast Cancer, Tianjin Medical University Cancer Institute and Hospital, Huan-Hu-Xi Road, He-Xi District, Tianjin, 300060 China; 7 Department of Hepatic Surgery, Fudan University Shanghai Cancer Center, Shanghai Medical College, Fudan University, Shanghai, 200032 China

**Keywords:** Krüppel‐like factors, Breast cancer, Bioinformatics, Prognosis

## Abstract

**Background:**

Krüppel‐like factors (KLFs) are zinc finger proteins which participate in transcriptional gene regulation. Although increasing evidence indicate that KLFs are involved in carcinogenesis and progression, its clinical significance and biological function in breast cancer are still limited.

**Methods:**

We investigated all the expression of KLFs (KLF1-18) at transcriptional levels by using Oncomine and Gene Expression Profiling Interactive Analysis (GEPIA). The mRNA and protein expression levels of KLFs were also determined by using RT-qPCR and immunohistochemistry, respectively. CBioPortal, GeneMANIA and STRING were used to comprehensive analysis of the molecular characteristics of KLFs. The clinical value of prognostic prediction based on the expression of KLFs was determined by using the KM plotter. The relevant molecular pathways of KLFs were further analyzed by using Gene Set Enrichment Analysis (GSEA) and Kyoto Encyclopedia of Genes and Genomes (KEGG) pathway database. Finally, we investigated the effect of KLF2 and KLF15 on biological behavior of breast cancer cells in vitro.

**Results:**

The expression of KLF2/4/6/8/9/11/15 was significantly down-regulated in breast cancer. The patients with high KLF2, KLF4 or KLF15 expression had a better outcome, while patients with high KLF8 or KLF11 had a poor prognosis. Furthermore, our results showed that KLF2 or KLF15 can be used as a prognostic factor independent on the other KLFs in patients with breast cancer. Overexpression of KLF2 or KLF15 inhibited cell proliferation and migration, and blocked cell cycle at G0/G1 phase, resulting in cell apoptosis.

**Conclusions:**

KLF2 and KLF15 function as tumor suppressors in breast cancer and are potential biomarkers for prognostic prediction in patients with breast cancer.

**Supplementary Information:**

The online version contains supplementary material available at 10.1186/s12935-022-02449-6.

## Introduction

Breast cancer has become the most commonly diagnosed malignancy worldwide (11.7% of total cases) and the leading cause of cancer death among women [1]. As a kind of highly heterogeneous tumor, breast cancer can be divided into 4 subtypes based on the gene expression profile and histological features, including luminal A, luminal B, HER2 enriched and basal-like subtypes [2]. Although recent advances in early diagnosis and systematic therapies have significantly improved the outcome in patients with breast cancer, patients with advanced stage breast cancer still suffer from local recurrence or distant metastasis [3]. Therefore, the identification of novel biomarkers and understanding of their mechanisms are useful for the diagnosis, treatment and prognostic prediction in patients with breast cancer.

The Krüppel-like factors (KLFs) belong to the zinc-finger family of transcription factors, which regulate diverse biological processes, including cell proliferation, differentiation, migration and survival [4]. 18 KLFs family members have been identified, all members contain three highly conserved Cys2/His2 zinc-fingers that facilitate binding to similar consensus sequences in regulatory regions of target genes. Increasing evidence indicates that the abnormal expression or activation of KLFs are associated with tumorigenesis and progression in almost all human cancers [5]. KLFs can function as both tumor suppressors or oncogenes, often with context-dependent functions depending on tissue, tumor type or cancer stage, implying their potential roles in predicting prognosis in different cancer patients [6]. Although several KLFs are closely implicated with cancer-related regulatory processes and signaling pathways involved in cell proliferation, apoptosis, invasion and migration [7, 8], the role and clinical value of KLFs in breast cancer are still unclear.

In this study, we performed integrated bioinformatic analysis to broadly investigate and obtain a deeper understanding of the relationship between KLFs and breast cancer. We determined the expression of KLFs at the mRNA and protein levels and evaluated the clinical prognostic value in patients with breast cancer. We further analyzed the genetic alteration, interaction network, and functional enrichment in breast cancer. Finally, in vitro experimental validation was confirmed KLF2 and KLF15 functioning as tumor suppressors, resulted in the inhibition of cell proliferation and migration in breast cancer. Our data will provide solid evidences for the prognostic prediction in patients with breast cancer.

## Materials and methods

### Tissue specimens

Twenty pairs of cancerous and adjacent non-cancerous breast tissues and paraffin-embedded tissue samples were collected at Tianjin Medical University Cancer Institute and Hospital. All tumor samples were histologically confirmed as breast cancer. This study was approved by the Tianjin Medical University Cancer Hospital Ethics Committee and was consistent with the ethical guidelines of the Helsinki Declaration.

### Oncomine analysis

Oncomine (https://www.Oncomine.org) is a public online cancer database for genome-wide expression analysis [9]. In the present study, we compared the transcriptional levels of KLFs in different types of cancer. The mRNA levels of KLFs in cancer samples were compared with those in normal samples using a student’s t test to generate a p value. Statistically significant values and fold change were defined as P-value < 0.01, and 2, respectively.

### GEPIA dataset analysis

Gene Expression Profiling Interactive Analysis (GEPIA) (http://gepia.cancer-pku.cn/), is a web application for analyzing RNA-seq data based on TCGA and GTEx data [10], which can be employed for multidimensional analysis. In this study, we utilized GEPIA to further investigate the correlation of gene expression and tissue type or clinical stages of the patients with breast cancer. The individualized studies were conducted under standard processing requirements.

### UALCAN analysis

UALCAN (http://ualcan.path.uab.edu) is an online comprehensive analysis of gene expression based on TCGA, which is aimed to identify tumor subgroup specific candidate biomarkers [11]. In this study, we further analyzed the relationship between the expression levels of different KLFs and the individual cancer stages via UALCAN database.

### RNA extraction, cDNA synthesis, and reverse transcription quantitative PCR (RT-qPCR)

TRIzol reagent (Ambion, USA) was used to extract the total RNA from surgically resected frozen breast tissues according to the manufacturer's guidelines. cDNA was synthesized by reverse transcription of RNA using PrimeScript RT Master Mix (TaKaRa, Japan). Quantitative RT-PCR (qPCR) was carried out using pre-designed primers according to the Manufacturer's instructions (Takara, Japan) with a Bio-Rad CFX96 system. The primers sequences were shown in Additional file 1: Table S1.

### Immunohistochemistry

Paraffin-embedded breast tissues were deparaffinized in xylene, rehydrated through an ethanol series, and antigen was then retrieved in citrate. Then, 3% H2O2 was used to block endogenous peroxidase activity for 15 min. Then samples were stained using antibodies at room temperature for 30 min and overnight at 4 °C. After washing, sections were incubated with secondary antibody PV-6001 kit (Zhongshan Biotechnology, China) at 37 °C for 1 h. The reaction product was developed using DAB detection kit (Zhongshan Biotechnology, China) and counterstained with hematoxylin. All images were captured with political fluorescence microscope.

Sections were scored blindly by two independent pathologists. The immunostaining percentage was scored as: 0 (0%), 1 (1%–25%), 2 (26%–50%), 3 (51%–75%), and 4 (> 75%); while staining intensity: 0 (negative staining), 1 (weak staining), 2 (moderate staining), and 3 (strong staining). The multiplier of the intensity and percentage scores was used as the final staining score.

### The Cancer Genome Atlas, and cBioPortal

The Cancer Genome Atlas (TCGA) is an open resource of cancer genomic profiles, which has sequencing, clinical information of patients with different types of cancers [12]. cBioPortal (www.cbioportal.org) is a comprehensive web resource for interactive exploration of multiple cancer genomic datasets [13]. Based on TCGA database, we used cBioPortal to investigate genomic profiles of KLFs, including mutations, copy number alterations (CNAs) and mRNA expression z-scores (RNA Seq V2 RSEM, ± 2).

### GeneMANIA and STRING analysis

GeneMANIA (http://genemania.org) is a web tool that provides information for protein and genetic interactions [14]. STRING (https://string-db.org/) is a protein Interaction (PPI) database used to predict physical interactions and functional correlations between proteins [15]. The interactions of KLFs at the gene and protein expression level were identified by using GeneMANIA and STRING.

### DAVID 6.8

DAVID 6.8 (https://david.ncifcrf.gov/home.jsp) is a comprehensive website for better elucidating the biological function of the submitted genes [16]. Based on DAVID 6.8, we proceed the Gene Ontology (GO) enrichment analysis and Kyoto Encyclopedia of Genes and Genomes (KEGG) pathway enrichment analysis of KLFs and closely related neighbor genes.Biological processes (BP), cellular components (CC), and molecular function (MF) were included in the GO enrichment analysis.

### The Kaplan–Meier plotter

The Kaplan–Meier plotter (http://kmplot.com/analysis/) was used to assess the clinical relationships between gene expression and survival information. In this study, samples were divided into high expression group and low expression group by the median expression level of KLFs.

### Cell culture

MCF10A, MCF-7, T47D, SK-BR-3, CAL-51 and MDA-MB-231cell lines were obtained from the Type Culture Collection of the Chinese Academy of Sciences. MDA-MB-231, CAL-51 and SKBR3 cell lines were cultured in RPMI-1640 medium (Gibco, USA) with 10% FBS (FBS; PAN-Seratech). MFC7 and T47D cell lines were cultured in DMEM medium (Gibco) with 10% FBS, while MCF10A cell lines were supplemented with MCF10A cell specific medium (DMEM/F12 + 5% HS + 20 ng/mL EGF + 0.5 μg/mL Hydrocortisone + 10 μg/mL Insulin + 1% NEAA + 1% P/S) (Procell, China). All the cells were supplemented with 1% penicillin–streptomycin (PS; HyClone), in a 5% CO2 and humidified atmosphere at 37 °C.

### Transfection

The KLF2 or KLF15 mammalian expression plasmid was bought from miaolingbio (Wuhan, China). For transfection, cells were plated at a density of 2 × 10^5^ cells well in six-well plates. When the cells were 60% confluent, 2 ug plasmid was transfected into cells using Lipofectamine 3000 (Invitrogen) according to the manufacturer’s recommendations. After the transfection, the RNA and protein were extracted.

### Western blot

Cells were washed with cold PBS three times and lysed on ice for 30 min using SDS lysis buffer supplemented with protease inhibitor cocktail (Roche, Switzerland). The collected protein was denatured in a 95 °C water bath for 10 min. Equal amounts of proteins (30 ug) were separated using SDS‐PAGE. Then, proteins were transferred to PVDF membranes and blocked with 5% bovine serum albumin, followed by incubation with primary and secondary antibodies. The details of the antibodies used in this study are shown in Additional file 1: Table S2.

### MTT assay

For MTT assay, 24 h after transfection, 5 × 10^3^ cells were plated in 96‐well plates and incubated for the appropriate time period. The cells were incubated with 20 μL MTT (5 mg/mL in PBS; Sigma) at 37 °C for 4 h. Then, the medium was removed and the formazan was dissolved in 150 μL of DMSO (Sigma). The absorbance at 570 nm was measured using a micro‐plate auto‐reader (Bio‐Rad).

### Colony Formation Assay

Cells (1000 cells/well) were seeded in 6-well plates in RPMI-1640 medium supplemented with 10% FBS. Two weeks later (when colonies had reached an appropriate size), the colony was rinsed 3 times with PBS, fixed with 4% paraformaldehyde for 10 min and subsequently stained with 0.5% crystal violet staining solution for 10 min. The colonies were photographed and counted under a microscope, and the data are presented as the means SDs of triplicate dishes in the same experiment.

### EdU assay

The EdU assay was detected by EdU labeling/detection kit (Ribobio) according to the manufacturer's protocol. Briefly, after transfection for 48 h, cells were incubated with 25 μM EdU for 12 h before fixation, permeabilization, and EdU staining. Subsequently, cell nuclei were stained with Hoechst 33,342 at a concentration of 5 μg/mL for 30 min. The percentage of EdU‐positive cells was examined by fluorescence microscopy.

### Transwell assay

RPMI-1640 supplemented with 10% FBS was loaded in the lower chamber, and 1 × 105 cells in serum-free RPMI-1640 were seeded on the 8-μm polyvinyl pyrrolidone-free polycarbonate filter membrane. After incubation for approximately 24 h in a 37 °C incubator, the migrated cells on the bottom were fixed and stained. Five random fields were captured by an optical microscope for cell quantification. All the measurements were detected in triplicate.

### Scratch assay

For the Scratch assay, 2 × 106 cells were plated in a 6-well plate. After the cells were confluent and attached, a 10 μL pipette tip was used to form a scratch mold in each well. The width of the wound was recorded at six random locations at the appropriate time points (0, 4, 8 and 12 h). Data are shown as the means and standard deviations (SDs). Images were acquired at 0, 4, 8 and 12 h.

### Gene Set Enrichment Analysis

Gene Set Enrichment Analysis (GSEA, https://www.gsea-msigdb.org/) is a computational method that assesses whether an a priori defined set of genes shows statistically significant, concordant differences between two biological states [17]. We used GSEA to explore pathways and gene sets associated with KLF2 and KLF15 in breast cancer. According to the order of expression level of KLF2 and KLF15 and the prognosis of cases, the optimal threshold in the ROC curve was divided into HIGH expression group and LOW expression group.

### Flow cytometric analysis

For cell cycle distribution, cells were digested and added to 95% ethanol at 4 °C overnight. Then the cells were stained with 500μL of propidium iodide (PI; BD Biosciences) after centrifugation and incubated in the dark for 15 min. The cell apoptosis assay was carried out according to the instructions of the APC Annexin V Apoptosis Detection Kit (BD Pharmingen). Cells were digested and stained with 5μL APC annexin V and 5μL PI in 1 × binding buffer for 15 min at room temperature in the dark. All samples were analyzed on a FACS Aria flow cytometer (BD) with CellQuest software, and the data were analyzed using FlowJo software.

## Results

### The expression of KLFs in breast cancer

**Fig. 1:**
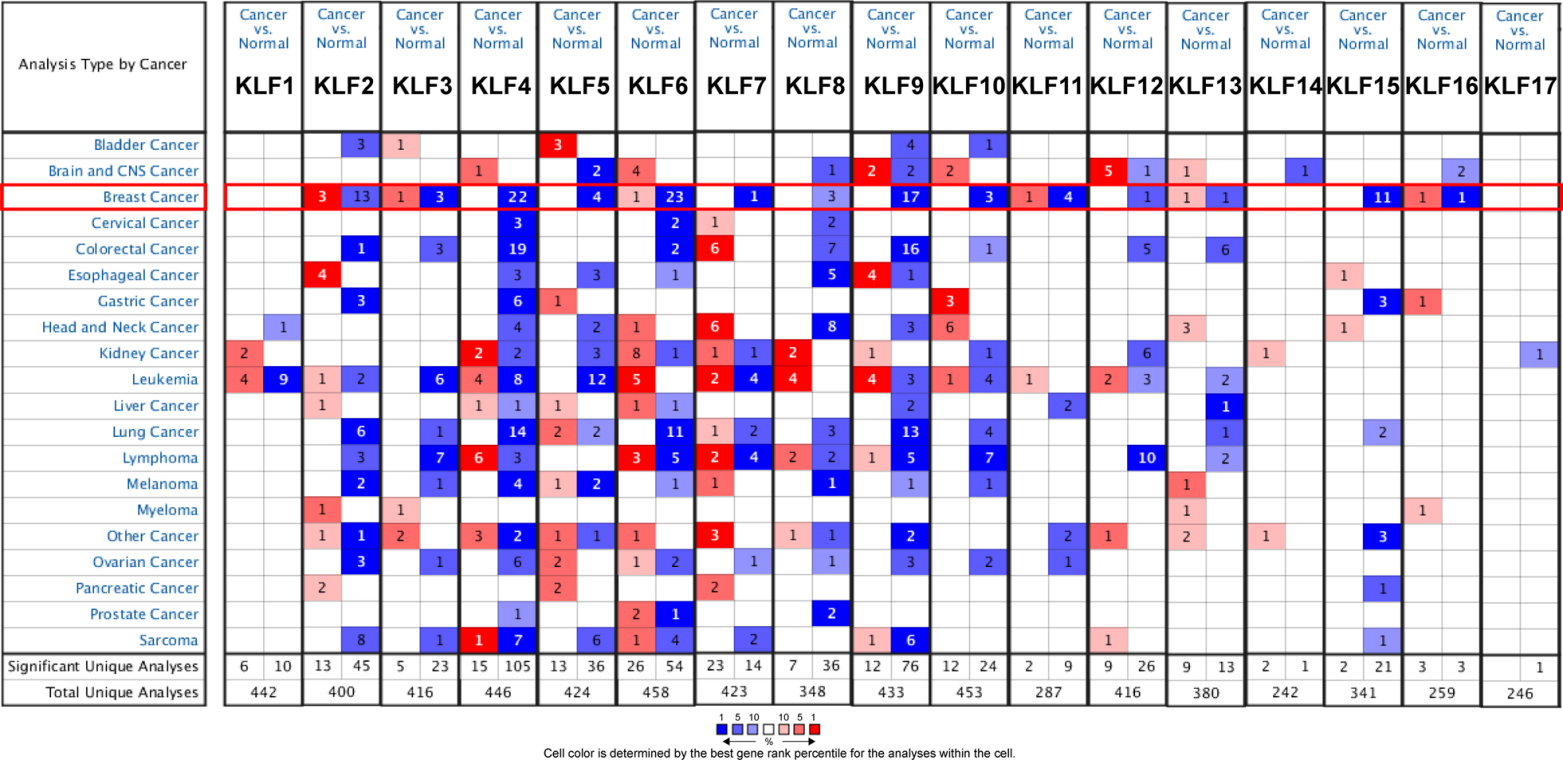
The transcription levels of KLFs in different types of cancers by Oncomine. Cancer vs. normal: up-regulated (red) or down-regulated (blue)

We used ONCOMINE database to search Seventeen KLFs (not excluding KLF18). The transcriptional levels of KLFs are presented in Fig. 1. In breast cancer, the mRNA expression levels of KLF4/5/7/8/9/10//12/15 were significantly down-regulated in multiple datasets. KLF2/3/6/11/13/16 has different expression levels among multiple data sets. The expression of KLF2 showed a significant upregulation in Invasive Breast Carcinoma Stroma, Invasive Ductal Breast Carcinoma Epithelia, Ductal Breast Carcinoma in Situ Epitheli, while other 13 dataset displayed an obvious downregulation across different subtypes. Only Finak Breast Statistics found that KLF3/6/11/16 was highly expressed in invasive breast carcinoma stroma, while KLF13 was higher in Invasive Ductal and Lobular Carcinoma. For KLF1/14/17, there was no significant difference between tumor tissues and normal tissues.

**Fig. 2:**
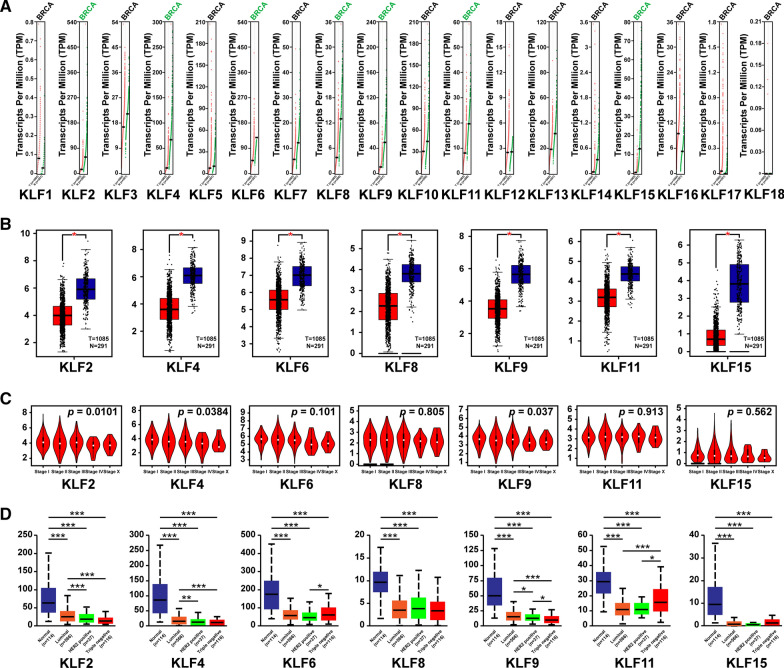
The expression levels of KLFs in breast cancer. **A**, **B** The expression of KLFs in breast cancer (T) and normal breast tissues (N) presented by scatter diagram (**A**) and box plot (**B**). **C** The relationship between KLFs expression levels and clinical stages of patients with breast cancer by GEPIA anslysis. **D** The relationship between KLFs expression levels and different molecular subtypes in breast cancer by UALCAN analysis. *P < 0.05, **P < 0.01, ***P < 0.001

We next compared the transcription levels of KLFs between breast cancer and normal tissues using GEPIA (Fig. 2A) and found that KLF2/4/6/8/9/11/15 were significantly downregulated in tumor tissues (Fig. 2B). We also analyzed the relationship between the mRNA levels of these KLFs and the tumor stages or molecular subtypes in breast cancer. The results revealed that the expression of KLF2/4/9 was associated with the tumor stage (Fig. 2C). In addition, we found that the expression of KLF2/4/9/11 have significant differences among different molecular subtypes by UALCAN (Fig. 2D).

**Fig. 3:**
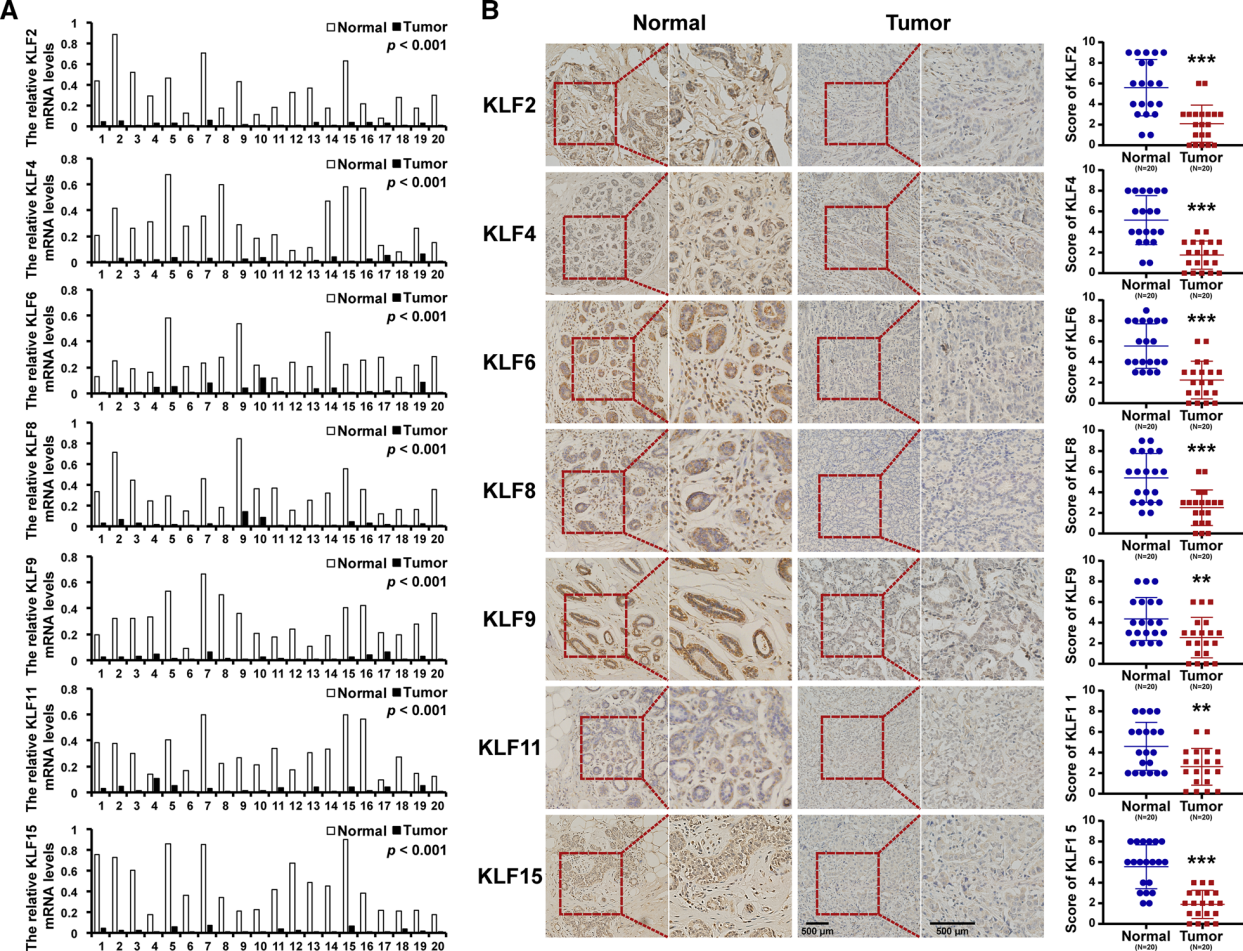
The expression of KLFs in breast cancer. **A** The mRNA expression levels of KLFs determined by RT-PCR. **B** The protein expression levels of KLFs determined by immunohistochemistry. **P < 0.01, ***P < 0.001

To validate these results from database, we performed RT-PCR and IHC analyses to determine the expression of these KLFs in breast cancer tissues and the paired normal breast tissues. Consistent with the database, the expression levels of KLF2/4/6/8/9/11/15 were significantly down-regulated in breast cancer tissues by RT-PCR and IHC (Fig. 3).

### Molecular characteristics of KLFs in breast cancer patients

**Fig. 4:**
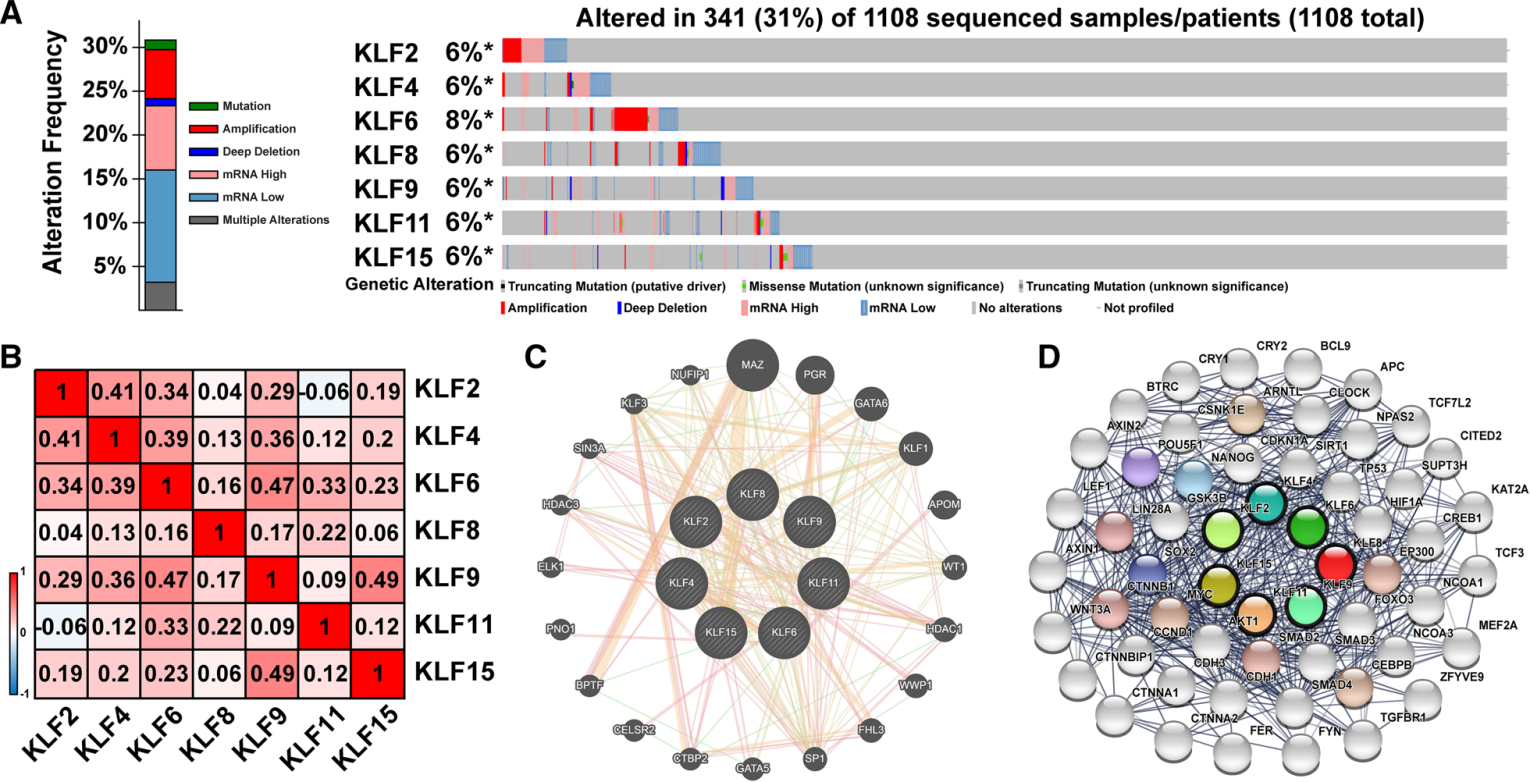
Molecular characteristics of KLFs in breast cancer patients. **A** Gene mutation analysis of KLFs in patients with breast cancer by cBioPortal analysis. **B** Pearson’s correlation analysis of KLFs. **C** GeneMANIA analysis of relevant interactive genes of KLFs. **D** Molecular network for KLFs and most frequently altered neighbor genes by STRING analysis

We investigated gene alterations, correlations and networks of above-mentioned differentially expressed KLFs in invasive breast cancer by using cBioPorta. Among the 7 KLFs, KLF6 achieved the highest alteration rate at 8%, of which the majority were amplification and mRNA dysregulation (Fig. 4A). Furthermore, the correlations of KLFs mRNA expression were calculated. The Spearman's correlation analysis indicated significant positive correlations between KLF9 and KLF6 or KLF15. In addition, a significant negative correlation was observed between KLF2 and KLF11(Fig. 4B). The molecular interaction network was then constructed for KLFs and other most frequently changed neighbor genes by using GeneMANIA and STRING database. We observed strong association between KLF2/9/15 and the most prominent weight of MAZ, PGR and GATA6 by using GeneMANIA (Fig. 4C). By using STRING database, we found that the KLFs were associated with Wnt signaling pathway-related genes, including AXIN1/2, WNT3A, CTNNB1, and cell cycle regulation-related genes, including CCND1, MYC, AKT1 (Fig. 4D).

### Prognostic values of KLFs in breast cancer patients

**Fig. 5:**
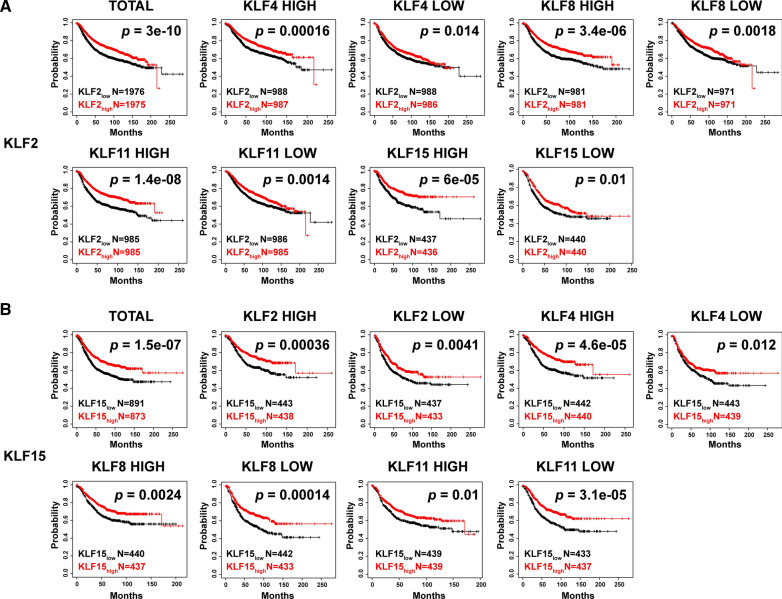
Cross-analysis of the RFS in patients with breast cancer based on KLFs expression levels by using KM plotter. **A** The association between KLF2 expression and RFS in patients with different KLFs levels analyzed by KM-plotter. **B** The association between KLF15 expression and RFS in patients with different KLFs levels analyzed by KM-plotter

Next, we investigated the associations between KLFs expression levels and prognosis in patients with breast cancer. We used KM plotter analysis to generate survival curve under multiple grouping condition. The distant metastasis-free survival (DMFS), relapse-free survival (RFS) and overall survival (OS) were summarized in Additional file 1: Fig. S1 and Table S4. We observed that the patients with high KLF2, KLF4 or KLF15 expression had a higher RFS than patients with low KLF2, KLF4 or KLF15 expression. In addition, the patients with high KLF8 or KLF11 expression had a lower OS, RFS and DMFS than patients with low KLF8 or KLF11 expression. However, the expression level of KLF6 or KLF9 was not associated with OS, RFS and DMFS in patients with breast cancer. We further cross-analyzed the RFS of patients with breast cancer based on KLF2/4/8/11/15 expression levels for intra-family study. Intra-family comparison showed no matter how much other KLFs expressed, both KLF2 (Fig. 5A) and KLF15 (Fig. 5B) expression levels can distinguish RFS of patients with breast cancer independent other KLFs expression levels (Additional file 1: Fig. S2). Taken together, these results suggest that KLF2 and KLF15 can be used as prognostic predictor in patients with breast cancer.

### KLF2 and KLF15 inhibit breast cancer cell proliferation and migration in vitro

**Fig. 6:**
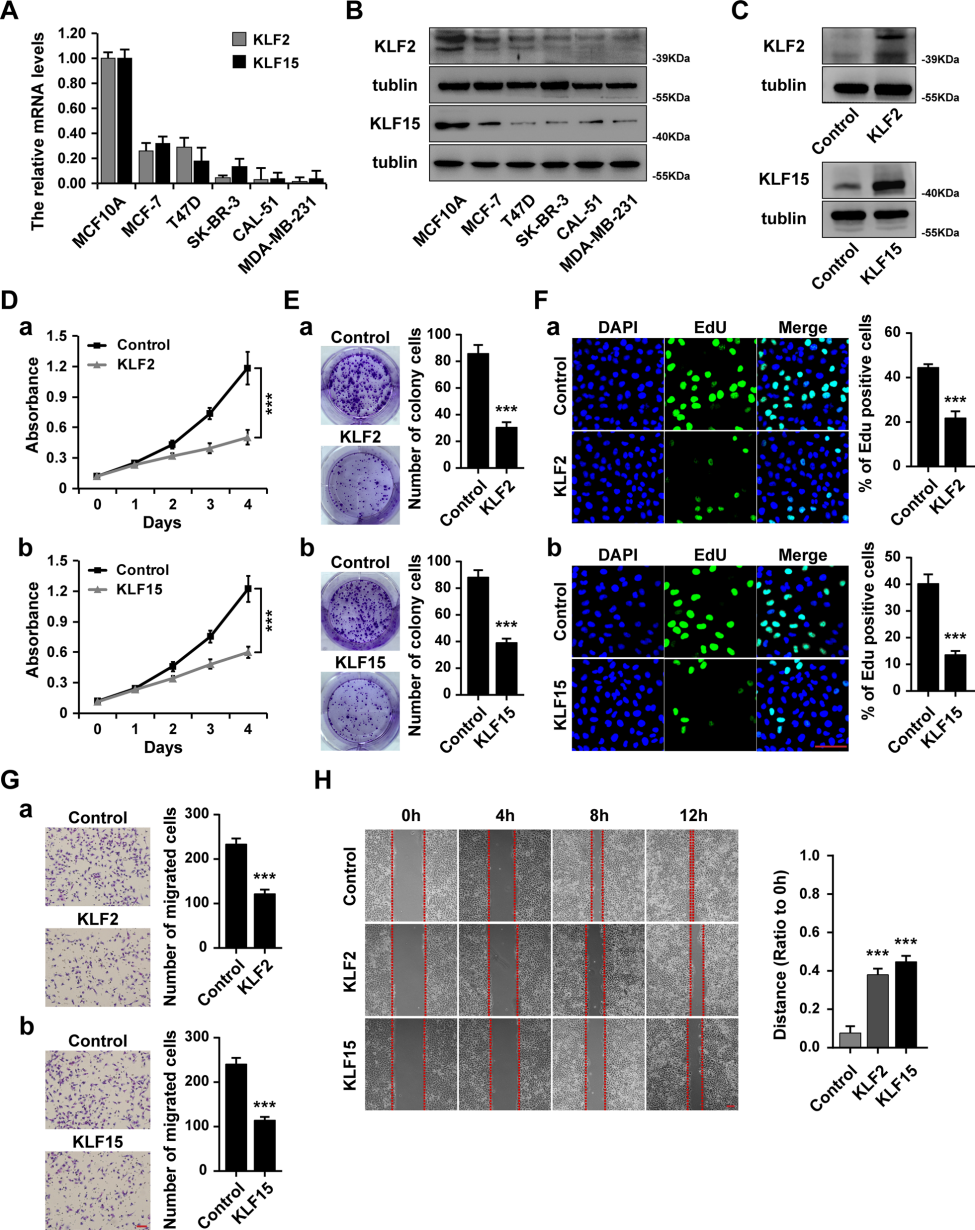
KLF2 and KLF15 inhibits breast cancer proliferation and migration in vitro. **A** The mRNA expression levels of KLF2 and KLF15 in breast cancer cell lines and normal breast cell line MCF10A determined by RT-qPCR. **B** The protein expression levels of KLF2 and KLF15 in breast cancer cell lines and normal breast cell line MCF10A determined by western blot. **C** The expression of KLF2 and KLF15 in KLF2 or KLF15 expressing plasmid transfected MDA-MB-231 cells determined by western blot. **D** Cell viability of KLF2-overexpressed (a) or KLF15-overexpressed (b) MDA-MB-231 cells, as well as control cells determined by MTT analysis. **E** Colony formation analysis of KLF2-overexpressed (a) or KLF15-overexpressed (b) MDA-MB-231 cells, as well as control cells. F, Edu analysis of KLF2-overexpressed (a) or KLF15-overexpressed (b) MDA-MB-231 cells, as well as control cells. **G** Transwell analysis of KLF2-overexpressed (a) or KLF15-overexpressed (b) MDA-MB-231 cells, as well as control cells. **H** Scratch analysis of KLF2-overexpressed or KLF15-overexpressed MDA-MB-231 cells, as well as control cells. ***P < 0.001

We next performed in vitro experiments to assess the effect of KLF2 and KLF15 on the biological behaviors in breast cancer cells. We firstly determined the expression levels of KLF2 and KLF15 in different breast cancer cell lines and the normal breast cell line by RT-PCR (Fig. 6A) and western blot (Fig. 6B). The results showed that both KLF2 and KLF15 expression was significantly lower in breast cancer cell lines, especially in the triple-negative breast cancer cell lines CAL51 and MDA-MB-231. Next, we assessed whether KLF2 or KLF15 overexpression in breast cancer cells can influence breast cancer proliferation and migration by KLF2- or KLF15-transfected MDA-MB-231 cells (Fig. 6C). The MTT and colony formation assays indicated that overexpression of KLF2 or KLF15 inhibited breast cancer cell proliferation (Fig. 6D, E). The EdU analysis also showed that the number of EdU-positive cells was significantly lower in KLF2- or KLF15-overexpressed MDA-MB-231 cells compared to the control cells (Fig. 6F). The transwell and scratch assays indicated that overexpression of KLF2 or KLF15 inhibited breast cancer cell migration (Fig. 6G, H)). Together, these results indicate that KLF2 and KLF15 function as tumor suppressors in breast cancer.

### KLF2 and KLF15 block cells at G1 phase and induce cell apoptosis

To obtain an in depth understanding the function of KLF2 and KLF15 in breast cancer progression, DAVID 6.8 was employed to analyze the functions of KLF2/15 and their neighboring genes. In gene ontology (GO) enrichment analysis, we observed that KLF2 or KLF15 alteration was associated with multiple biological processes, including cell proliferation (GO:0008283), cell adhesion (GO:0007155), regulation of cell growth (GO:0001558) and regulation of cell cycle (GO:0051726) in breast cancer (Additional file 1: Fig. S3A). We next performed KEGG analysis to explore the pathways associated with KLF2 and KLF15 altered functions and neighbor genes. As shown in Additional file 1: Fig. S3B, cell cycle, pathways in cancer, PI3K-Akt signaling pathway and PPAR signaling pathway were significantly involved in breast tumorigenesis and progression.

**Fig. 7:**
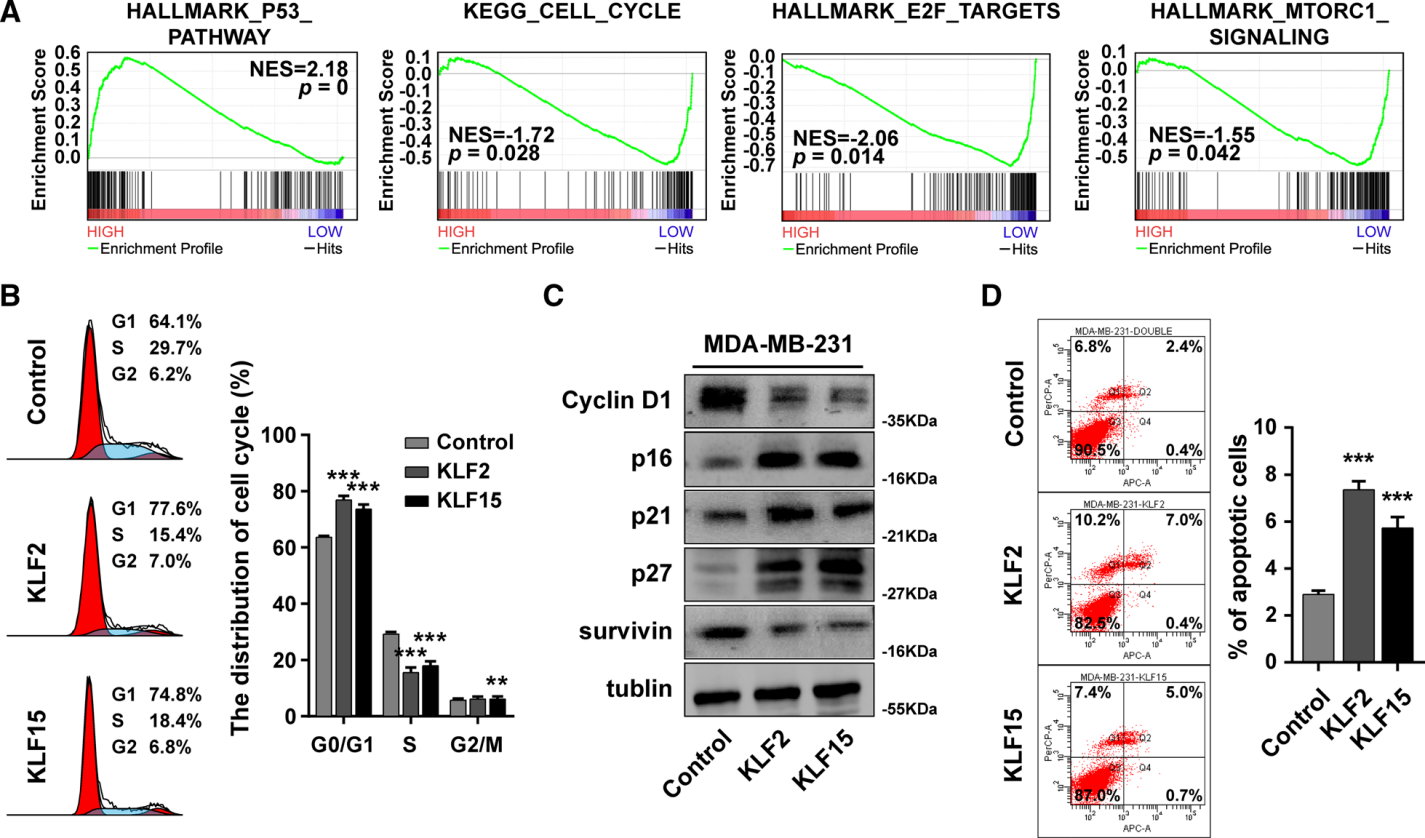
KLF2 and KLF15 induce cell apoptosis and cell cycle arrest at G1 phase. **A** GSEA analysis of KLF2/15 expression levels and relevant signaling pathways. **B** The cell cycle distribution of KLF2 or KLF15-overexpressed MDA-MB-231 and control cells determined by flow cytometry analysis. **C** The expression of Cyclin D1, survivin, p16, p21 and p27 determined by western blot. **D** The cell apoptosis analysis of KLF2 or KLF15-overexpressed MDA-MB-231 and control cells determined by flow cytometry analysis. ***P < 0.001

We next employed GSEA analysis to explore the relevant signaling pathways of KLF2 and KLF15 high expression group and low expression group in patients with breast cancer. The results indicated that KLF2 and KLF15 alteration were significantly association with several cancer-related pathways, including P53 pathway, Cell Cycle, E2F targets and mTORC1 signaling (Fig. 7A). Furthermore, overexpression of KLF2 or KLF15 induced cell cycle arrest at G1 phase (Fig. 7B). In addition, the expression of cell cycle inhibitor p16, p21 and p27 was raised, whereas the expression of Cyclin D1 and surviving was reduced in KLF2- or KLF15-overexpressed MDA-MB-231 by western blot (Fig. 7C). The number of apoptotic cells was significantly higher in KLF2- or KLF15-overexpressed MDA-MB-231 compared with the control cells (Fig. 7D). Collectively, these results indicate that KLF2 and KLF15 induce cell cycle arrest and cell apoptosis in breast cancer.

## Discussion

In the current study, we aimed to investigate the role of KLFs in breast cancer progression and found that the expression of KLF2 and KLF15 can be used as the prognostic predictor in patients with breast cancer. Integrated bioinformatic analysis indicated that the expression of KLF2 and KLF15 is closely associated with cell cycle progression. Moreover, overexpression of KLF2 and KLF15 induced cell cycle arrest at G1 phase, resulting in inhibition of proliferation and induction of apoptosis.

KLFs belong to a zinc-finger transcription factor family, which is involved in various cellular biological processes and diverse human diseases, including cancers [6, 8, 18, 19]. Recently, increasing evidence suggests that several KLFs participate in multiple biological processes during breast cancer progression, including proliferation, migration, invasion, metastasis, apoptosis and stemness [20]. Consistent with previous study, we observed that the expression of KLF2/4/6/8/9/11/15 is down-regulated in breast cancer tissues compared to normal breast tissues [21]. Although the prognostic signatures based on KLFs expression have been investigated in several studies, we found that both KLF2 and KLF15 can be used as prognostic predictors independent on other KLFs in patients with breast cancer [21, 22]. Furthermore, we demonstrated that KLF2 and KLF15 can function as tumor suppressors in breast cancer.

Dysregulation of KLF2 and KLF15 has been reported to play vital roles in a variety of human cancers [23, 24]. Consistent with previous study, we demonstrated that KLF2 and KLF15 inhibits breast cancer proliferation and migration [25–28]. GSEA analysis further revealed that abnormal KLF2 and KLF15 expression is closely associated with cell cycle-related genes. Previous studies indicated that KLF2 significantly suppresses tumor cell viability and induces cell cycle arrest at G0/G1 through up-regulation of p15 and p21 expression in non-small cell lung cancer [29]. Moreover, KLF15 suppressed breast cancer cell proliferation and induced cell cycle arrest by up-regulation of p21 [30]. In current study, we observed that overexpression of KLF2 or KLF15 induces cell apoptosis and cell cycle arrest at G1 phase. The cell cycle inhibitors p16, p21 and p27 were increased in KLF2- or KLF15-overexpressed cells, whereas the cyclin D1 and survivin were under-expressed in those cells, suggesting that both KLF2 and KLF15 are negative regulators of cell cycle progression.

## Conclusions

In summary, the dysregulation expression of KLF2 and KLF15 in breast cancer tissues might play an important role in BC oncogenesis. Our study demonstrated that KLF2 and KLF15 function as tumor suppressors, may serve as prognostic biomarkers in patients with breast cancer.

## Supplementary Information

**Additional file 1.** Supplementary information.

## Data Availability

All data generated or analyzed during this study are included in this published article and its additional file.
