# Supplementary material for: The functions and prognostic value of Krüppel‐like factors in breast cancer
Source: Cancer Cell Int. 2022 Jan 15;22:23. doi: 10.1186/s12935-022-02449-6 (PMC8760734; doi:10.1186/s12935-022-02449-6)
Supplement: Supplementary file 1 — Additional file 1. Supplementary information. [file 12935_2022_2449_MOESM1_ESM.docx]

**
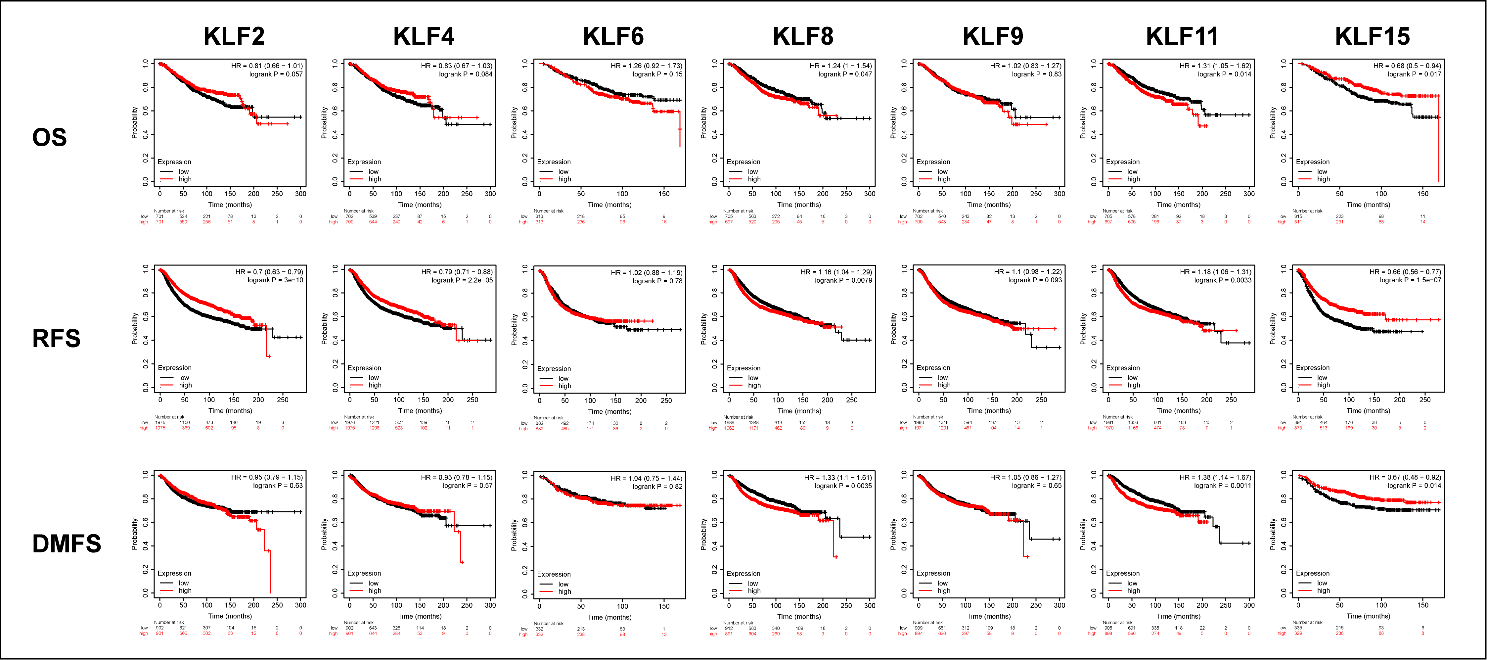
**

**Figure S1. The association of KLFs expression levels with outcomes in patients with breast cancer by KM-plotter.**

**
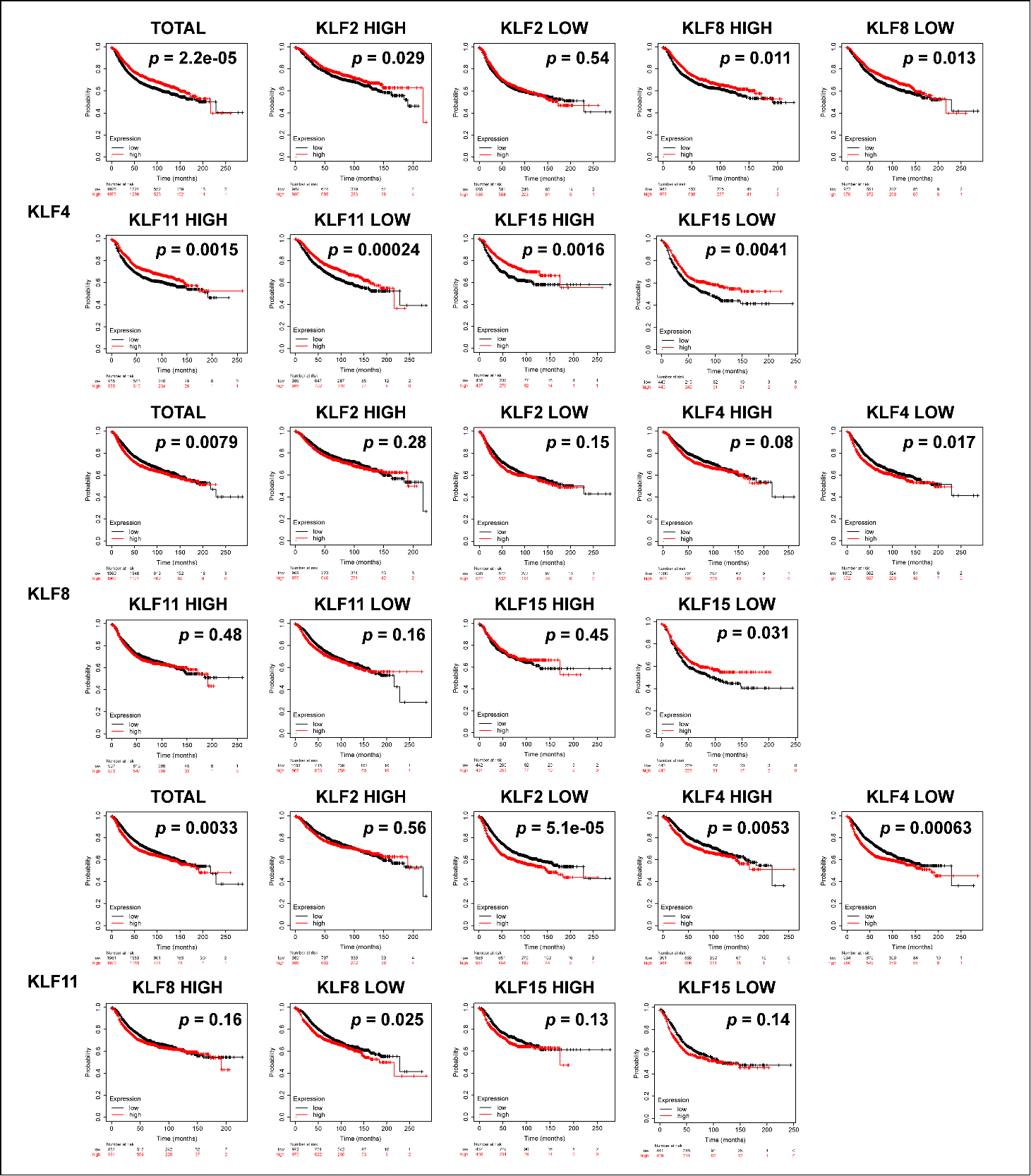
**

**Figure S2. Cross-analysis of the RFS in patients with breast cancer based on KLFs expression levels by using KM plotter.**

**
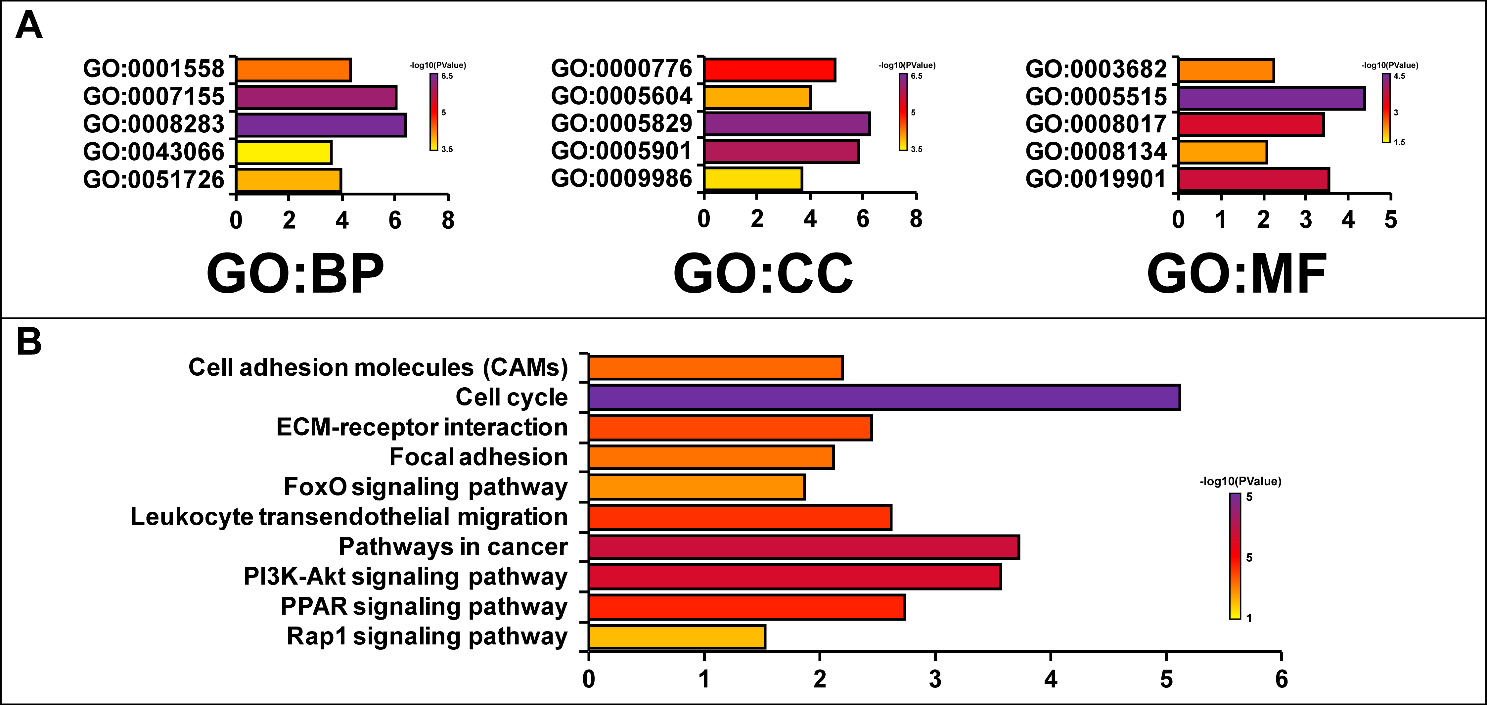
**

**Figure S3. The enrichment analysis of KLF2/15 and 50 most frequently altered neighboring genes. A,** Gene significantly associated with KLF2 and KLF15 alteration by GO enrichment analysis. Biological processes (BP), cellular components (CC) and molecular functions (MF). **B,** Molecular pathways associated with KLF2 and KLF15 regulation by KEGG analysis.

**Table S1. Primers sequences used for RT-PCR**

| **Gene name** | **Sequences (5’ to 3’)** |
| --- | --- |
| KLF2 | 5’-CTGCACATGAAACGGCACAT-3’ |
|  | 5’-CAGTCACAGTTTGGGAGGGG-3’ |
| KLF4 | 5’-TCCAAAGAAGAAGGATCTCGGCCA-3’ |
|  | 5’-AACGTGGAGAAAGATGGGAGCA-3’ |
| KLF6 | 5’-GGCAACAGACCTGCCTAGAG-3’ |
|  | 5’-CTCCCGAGCCAGAATGATTTT-3’ |
| KLF8 | 5’-CCCAAGTGGAACCAGTTGACC-3’ |
|  | 5’-GACGTGGACACCACAAGGG-3’ |
| KLF9 | 5’-GGGAAACCTCCGAAAA-3’ |
|  | 5’-CGTTCACCTGTATGCACTGTA-3’ |
| KLF11 | 5’-ACGGTCTTGGCGGCCTAG-3’ |
|  | 5’-ACTTTCATCAAAACCAGCCTCC-3’ |
| KLF15 | 5’-TCTCGC TGGAGGCCAGG-3’ |
|  | 5’-TCCATCACCCGGCAGGA-3’ |
| GAPDH | 5’-GTCTCCTCTGACTTCAACAGCG-3’ |
|  | 5’-ACCACCCTGTTGCTGTAGCCAA-3’ |

**Table S2. Antibodies used for study**

| Antibody | Company |
| --- | --- |
| KLF-2 (bs-2772R) | Bioss Antibodies |
| KLF-4 (bs-1064R) | Bioss Antibodies |
| KLF-6 (bs-1395R) | Bioss Antibodies |
| KLF-9 (bs-3644R) | Bioss Antibodies |
| KLF-11(YT4653) | ImmunoWay Biotechnology |
| KLF-15 (A-5, sc-271675) | Santa Cruz Biotechnology |
| Cyclin D1 (DCS-6, sc-20044) | Santa Cruz Biotechnology |
| P16 (554079) | BD PharMingen |
| P21 (F-5, sc-6246) | Santa Cruz Biotechnology |
| P27 (3686T) | Cell Signaling Technology |
| survivin (2808T) | Cell Signaling Technology |
| tublin (66031-1-Ig) | Proteintech |

**Table S3. The Significant Changes of KLFs Expression in Transcription Level between Different Types of Breast Cancer and Normal Breast Tissues (Oncomine Database)**

| KLF2 | Type of Breast Cancer versus Normal Breast Tissue | | Fold Change | p Value | t Test | Source and/or Reference |
| --- | --- | --- | --- | --- | --- | --- |
|  | Breast Carcinoma | -3.474 | | 6.59E-10 | -11.972 | Curtis Breast Statistics[1] |
|  | Invasive Ductal Breast Carcinoma | -3.104 | | 2.70E-72 | -29.931 | Curtis Breast Statistics[1] |
|  | Breast Phyllodes Tumor | -4.431 | | 1.84E-04 | -9.669 | Curtis Breast Statistics[1] |
|  | Medullary Breast Carcinoma | -3.9 | | 5.54E-17 | -13.596 | Curtis Breast Statistics[1] |
|  | Invasive Breast Carcinoma | -2.953 | | 7.01E-09 | -8.452 | Curtis Breast Statistics[1] |
|  | Tubular Breast Carcinoma | -2.76 | | 1.14E-28 | -14.746 | Curtis Breast Statistics[1] |
|  | Invasive Lobular Breast Carcinoma | -2.151 | | 2.52E-34 | -13.943 | Curtis Breast Statistics[1] |
|  | Invasive Ductal and Invasive Lobular Breast Carcinoma | -2.298 | | 1.11E-28 | -13.363 | Curtis Breast Statistics[1] |
|  | Mucinous Breast Carcinoma | -2.806 | | 9.85E-17 | -11.165 | Curtis Breast Statistics[1] |
|  | Mixed Lobular and Ductal Breast Carcinoma | -2.059 | | 3.45E-05 | -5.077 | TCGA Breast Statistics |
|  | Invasive Breast Carcinoma | -3.549 | | 1.54E-13 | -8.088 | TCGA Breast Statistics |
|  | Invasive Ductal Breast Carcinoma | -3.782 | | 1.46E-19 | -11.065 | TCGA Breast Statistics |
|  | Invasive Ductal Breast Carcinoma | -2.427 | | 3.00E-03 | -3.136 | Radvanyi Breast Statistics[2] |
|  | Invasive Breast Carcinoma Stroma | 37.415 | | 7.60E-29 | 22.739 | Finak Breast Statistics[3] |
|  | Invasive Ductal Breast Carcinoma Epithelia | 2.833 | | 3.00E-03 | 3.268 | Ma Breast 4 Statistics[4] |
|  | Ductal Breast Carcinoma in Situ Epitheli | 2.307 | | 5.00E-03 | 2.985 | Ma Breast 4 Statistics[4] |
| KLF3 | Invasive Ductal Breast Carcinoma | -3.65 | | 1.92E-05 | -4.95 | Radvanyi Breast Statistics[2] |
|  | Invasive Lobular Breast Carcinoma | -5.89 | | 2.00E-03 | -4.57 | Radvanyi Breast Statistics[2] |
|  | Mucinous Breast Carcinoma | -2.363 | | 2.00E-03 | -5.119 | TCGA Breast Statistics |
|  | Invasive Breast Carcinoma Stroma | 2.533 | | 4.00E-19 | 13.271 | Finak Breast Statistics[3] |
| KLF4 | Lobular Breast Carcinoma | -4.75 | | 8.17E-04 | -4.788 | Sorlie Breast 2 Statistics[5] |
|  | Ductal Breast Carcinoma | -5.482 | | 2.57E-05 | -13.089 | Sorlie Breast 2 Statistics[5] |
|  | Lobular Breast Carcinoma | -6.721 | | 2.00E-03 | -6.283 | Perou Breast Statistics[6] |
|  | Ductal Breast Carcinoma | -5.365 | | 3.62E-05 | -11.901 | Perou Breast Statistics[6] |
|  | Ductal Breast Carcinoma | -5.529 | | 1.36E-06 | -13.094 | Sorlie Breast Statistics[7] |
|  | Invasive Ductal Breast Carcinoma | -2.115 | | 2.99E-09 | -7.759 | Zhao Breast Statistics[8] |
|  | Medullary Breast Carcinoma | -7.977 | | 3.32E-30 | -20.809 | Curtis Breast Statistics[1] |
|  | Invasive Lobular Breast Carcinoma | -4.005 | | 9.72E-47 | -17.268 | Curtis Breast Statistics[1] |
|  | Invasive Breast Carcinoma | -4.256 | | 2.53E-11 | -10.287 | Curtis Breast Statistics[1] |
|  | Invasive Ductal Breast Carcinoma | -5.629 | | 1.36E-68 | -29.874 | Curtis Breast Statistics[1] |
|  | Invasive Ductal and Invasive Lobular Breast Carcinoma | -3.765 | | 8.22E-35 | -15.068 | Curtis Breast Statistics[1] |
|  | Tubular Breast Carcinoma | -3.778 | | 6.15E-31 | -14.697 | Curtis Breast Statistics[1] |
|  | Breast Carcinoma | -4.01 | | 1.90E-07 | -8.153 | Curtis Breast Statistics[1] |
|  | Mucinous Breast Carcinoma | -4.522 | | 8.88E-18 | -11.524 | Curtis Breast Statistics[1] |
|  | Ductal Breast Carcinoma | -3.454 | | 1.36E-04 | -5.428 | Curtis Breast Statistics[1] |
|  | Mucinous Breast Carcinoma | -6.246 | | 2.34E-05 | -8.814 | TCGA Breast Statistics |
|  | Male Breast Carcinoma | -4.228 | | 4.65E-05 | -8.016 | TCGA Breast Statistics |
|  | Invasive Lobular Breast Carcinoma | -3.605 | | 3.85E-11 | -7.343 | TCGA Breast Statistics |
|  | Invasive Breast Carcinoma | -3.249 | | 6.11E-16 | -9.246 | TCGA Breast Statistics |
|  | Mixed Lobular and Ductal Breast Carcinoma | -4.05 | | 1.38E-04 | -5.329 | TCGA Breast Statistics |
|  | Invasive Ductal Breast Carcinoma | -4.148 | | 1.44E-21 | -13.354 | TCGA Breast Statistics |
|  | Invasive Ductal Breast Carcinoma Stroma | -2.449 | | 2.00E-03 | -3.522 | Karnoub Breast Statistics[9] |
| KLF5 | Ductal Breast Carcinoma in Situ Epithelia | -4.478 | | 3.15E-06 | -6.098 | Ma Breast 4 Statistics[4] |
|  | Invasive Ductal Breast Carcinoma Epithelia | -3.708 | | 1.36E-05 | -5.567 | Ma Breast 4 Statistics[4] |
|  | Invasive Breast Carcinoma Stroma | -16.782 | | 1.21E-28 | -20.867 | Finak Breast Statistics[3] |
|  | Invasive Ductal Breast Carcinoma | -2.399 | | 1.00E-03 | -3.341 | Radvanyi Breast Statistics[2] |
| KLF6 | Ductal Breast Carcinoma | -3.134 | | 1.46E-10 | -13.696 | Perou Breast Statistics[6] |
|  | Lobular Breast Carcinoma | -3.132 | | 5.22E-04 | -5.18 | Sorlie Breast 2 Statistics[5] |
|  | Ductal Breast Carcinoma | -2.693 | | 2.89E-04 | -8.048 | Sorlie Breast 2 Statistics[5] |
|  | Lobular Breast Carcinoma | -3.136 | | 2.00E-03 | -4.45 | Sorlie Breast Statistics[7] |
|  | Fibroadenoma | -2.211 | | 9.00E-03 | -3.496 | Sorlie Breast Statistics[7] |
|  | Ductal Breast Carcinoma | -2.795 | | 2.66E-04 | -7.194 | Sorlie Breast Statistics[7] |
|  | Invasive Lobular Breast Carcinoma | -3.067 | | 5.36E-63 | -22.433 | Curtis Breast Statistics[1] |
|  | Invasive Ductal Breast Carcinoma | -3.467 | | 5.82E-98 | -40.405 | Curtis Breast Statistics[1] |
|  | Tubular Breast Carcinoma | -3.58 | | 1.09E-36 | -19.886 | Curtis Breast Statistics[1] |
|  | Invasive Ductal and Invasive Lobular Breast Carcinoma | -2.861 | | 1.19E-38 | -17.903 | Curtis Breast Statistics[1] |
|  | Breast Carcinoma | -3.23 | | 4.20E-08 | -9.797 | Curtis Breast Statistics[1] |
|  | Mucinous Breast Carcinoma | -3.371 | | 4.96E-19 | -13.252 | Curtis Breast Statistics[1] |
|  | Medullary Breast Carcinoma | -3.192 | | 3.53E-12 | -10.06 | Curtis Breast Statistics[1] |
|  | Invasive Breast Carcinoma | -3.178 | | 1.75E-06 | -6.237 | Curtis Breast Statistics[1] |
|  | Ductal Breast Carcinoma in Situ | -2.516 | | 1.78E-04 | -5.403 | Curtis Breast Statistics[1] |
|  | Breast Phyllodes Tumor | -2.611 | | 8.00E-03 | -3.907 | Curtis Breast Statistics[1] |
|  | Intraductal Cribriform Breast Adenocarcinoma | -2.064 | | 2.42E-10 | -8.456 | TCGA Breast Statistics |
|  | Mucinous Breast Carcinoma | -2.486 | | 5.64E-06 | -8.715 | TCGA Breast Statistics |
|  | Mixed Lobular and Ductal Breast Carcinoma | -2.068 | | 1.05E-06 | -7.639 | TCGA Breast Statistics |
|  | Invasive Lobular Breast Carcinoma | -2.023 | | 1.87E-09 | -6.641 | TCGA Breast Statistics |
|  | Invasive Ductal Breast Carcinoma | -2.206 | | 1.69E-21 | -12.884 | TCGA Breast Statistics |
|  | Invasive Ductal Breast Carcinoma | -2.023 | | 3.00E-03 | -5.248 | Zhao Breast Statistics[8] |
|  | Invasive Breast Carcinoma | -2.983 | | 1.00E-03 | -8.409 | Glück Breast Statistics[10] |
|  | Invasive Breast Carcinoma Stroma | 5.69 | | 5.41E-13 | 14.264 | Finak Breast Statistics[3] |
| KLF7 | Fibroadenoma | -2.037 | | 2.00E-03 | -6.526 | Sorlie Breast 2 Statistics[5] |
| KLF8 | Intraductal Cribriform Breast Adenocarcinoma | -2.728 | | 2.00E-03 | -7.357 | TCGA Breast Statistics |
|  | Invasive Lobular Breast Carcinoma | -2.292 | | 3.90E-09 | -6.578 | TCGA Breast Statistics |
|  | Invasive Ductal Breast Carcinoma | -2.601 | | 2.50E-20 | -11.389 | TCGA Breast Statistics |
| KLF9 | Tubular Breast Carcinoma | -2.544 | | 9.35E-43 | -18.97 | Curtis Breast Statistics[1] |
|  | Invasive Ductal Breast Carcinoma | -3.317 | | 2.29E-84 | -35.46 | Curtis Breast Statistics[1] |
|  | Invasive Lobular Breast Carcinoma | -2.573 | | 1.78E-49 | -18.221 | Curtis Breast Statistics[1] |
|  | Medullary Breast Carcinoma | -4.559 | | 8.20E-18 | -14.959 | Curtis Breast Statistics[1] |
|  | Mucinous Breast Carcinoma | -3.024 | | 1.33E-23 | -15.119 | Curtis Breast Statistics[1] |
|  | Invasive Ductal and Invasive Lobular Breast Carcinoma | -2.84 | | 2.33E-34 | -16.089 | Curtis Breast Statistics[1] |
|  | Invasive Breast Carcinoma | -2.568 | | 1.29E-09 | -9.113 | Curtis Breast Statistics[1] |
|  | Ductal Breast Carcinoma in Situ | -2.15 | | 1.42E-05 | -6.939 | Curtis Breast Statistics[1] |
|  | Breast Carcinoma | -2.986 | | 1.71E-06 | -7.32 | Curtis Breast Statistics[1] |
|  | Male Breast Carcinoma | -2.258 | | 1.63E-10 | -7.954 | TCGA Breast Statistics |
|  | Invasive Breast Carcinoma | -3.322 | | 9.73E-24 | -12.172 | TCGA Breast Statistics |
|  | Invasive Ductal Breast Carcinoma | -4.088 | | 3.21E-32 | -19.078 | TCGA Breast Statistics |
|  | Mixed Lobular and Ductal Breast Carcinoma | -3.855 | | 1.81E-05 | -7.772 | TCGA Breast Statistics |
|  | Intraductal Cribriform Breast Adenocarcinoma | -4.024 | | 8.32E-04 | -7.786 | TCGA Breast Statistics |
|  | Invasive Lobular Breast Carcinoma | -2.626 | | 4.09E-10 | -7.282 | TCGA Breast Statistics |
|  | Invasive Ductal Breast Carcinoma Stroma | -2.507 | | 1.76E-04 | -4.97 | Karnoub Breast Statistics[9] |
|  | Invasive Breast Carcinoma | -4.854 | | 2.78E-04 | -12.087 | Glück Breast Statistics[10] |
| KLF10 | Mucinous Breast Carcinoma | -3.594 | | 7.60E-22 | -16.652 | TCGA Breast Statistics |
|  | Invasive Lobular Breast Carcinoma | -2.004 | | 2.61E-11 | -7.427 | TCGA Breast Statistics |
|  | Invasive Breast Carcinoma | -3.006 | | 3.00E-03 | -6.47 | Glück Breast Statistics[10] |
| KLF11 | Lobular Breast Carcinoma | -2.326 | | 8.19E-06 | -5.852 | Zhao Breast Statistics[8] |
|  | Invasive Ductal Breast Carcinoma | -2.844 | | 3.90E-07 | -11.003 | Zhao Breast Statistics[8] |
|  | Mixed Lobular and Ductal Breast Carcinoma | -2.234 | | 1.41E-08 | -9.836 | TCGA Breast Statistics |
|  | Invasive Breast Carcinoma | -2.175 | | 9.31E-04 | -7.886 | Glück Breast Statistics[10] |
|  | Invasive Breast Carcinoma Stroma | 2.097 | | 4.47E-21 | 14.502 | Finak Breast Statistics[3] |
| KLF12 | Mucinous Breast Carcinoma | -3.187 | | 6.41E-04 | -7.663 | TCGA Breast Statistics |
| KLF13 | Mucinous Breast Carcinoma | -2.883 | | 2.69E-04 | -9.667 | TCGA Breast Statistics |
|  | Invasive Ductal and Lobular Carcinoma | 2.632 | | 5.63E-04 | 4.55 | TCGA Breast Statistics |
| KLF15 | Invasive Ductal Breast Carcinoma Stroma | -2.948 | | 6.34E-07 | -7.042 | Karnoub Breast Statistics[9] |
|  | Invasive Mixed Breast Carcinoma | -3.286 | | 7.04E-04 | -4.795 | Radvanyi Breast Statistics[2] |
|  | Invasive Ductal Breast Carcinoma | -7.399 | | 7.47E-06 | -5.688 | Radvanyi Breast Statistics[2] |
|  | Ductal Breast Carcinoma in Situ | -3.26 | | 8.75E-04 | -5.331 | Radvanyi Breast Statistics[2] |
|  | Invasive Lobular Breast Carcinoma | -4.432 | | 5.00E-03 | -3.467 | RadvanyiBreast Statistics[2] |
|  | Invasive Breast Carcinoma | -6.216 | | 1.24E-28 | -14.319 | TCGA Breast Statistics |
|  | Invasive Lobular Breast Carcinoma | -5.008 | | 5.90E-16 | -10.183 | TCGA Breast Statistics |
|  | Intraductal Cribriform Breast Adenocarcinoma | -17.473 | | 1.20E-05 | -14.244 | TCGA Breast Statistics |
|  | Invasive Ductal Breast Carcinoma | -14.648 | | 3.92E-33 | -20.206 | TCGA Breast Statistics |
|  | Invasive Ductal and Lobular Carcinoma | -11.491 | | 1.49E-04 | -10.992 | TCGA Breast Statistics |
|  | Mucinous Breast Carcinoma | -7.523 | | 2.00E-03 | -5.997 | TCGA Breast Statistics |
| KLF16 | Invasive Breast Carcinoma | -3.198 | | 6.02E-36 | -23.437 | Glück Breast Statistics[10] |
|  | Invasive Breast Carcinoma Stroma | 2.226 | | 1.54E-18 | 15.07 | Finak Breast Statistics[3] |

**Table S4. The prognostic value of KLFs in patients with breast cancer**

| Gene | Dataset/  Affymetrix ID | Survival outcome | No.of cases | HR | 95% CI | *p*-value |
| --- | --- | --- | --- | --- | --- | --- |
| *KLF2* | 219371_s_at | OS | 1402 | 0.81 | 0.66-1.01 | 0.057 |
|  |  | RFS | 3951 | 0.7 | 0.63-0.79 | **3.00E-10** |
|  |  | DMFS | 1803 | 0.95 | 0.79-1.15 | 0.63 |
| *KLF4* | 221841_s_at | OS | 1402 | 0.83 | 0.67-1.03 | 0.084 |
|  |  | RFS | 3951 | 0.79 | 0.71-0.88 | **2.20E-05** |
|  |  | DMFS | 1803 | 0.95 | 0.78-1.15 | 0.57 |
| *KLF6* | 1555832_s_at | OS | 626 | 1.26 | 0.92-1.73 | 0.15 |
|  |  | RFS | 1764 | 1.02 | 0.88-1.19 | 0.78 |
|  |  | DMFS | 664 | 1.04 | 0.75-1.44 | 0.82 |
| *KLF8* | *219930_at* | OS | 1402 | 1.24 | 1-1.54 | **0.047** |
|  |  | RFS | 3951 | 1.16 | 1.04-1.29 | **0.0079** |
|  |  | DMFS | 1803 | 1.33 | 1.1-1.61 | **0.0035** |
| *KLF9* | 203543_s_at | OS | 1402 | 1.02 | 0.83-1.27 | 0.83 |
|  |  | RFS | 3951 | 1.1 | 0.98-1.22 | 0.093 |
|  |  | DMFS | 1803 | 1.05 | 0.86-1.27 | 0.65 |
| *KLF11* | 218486_at | OS | 1402 | 1.31 | 1.05-1.62 | **0.014** |
|  |  | RFS | 3951 | 1.18 | 1.06-1.31 | **0.0033** |
|  |  | DMFS | 1803 | 1.38 | 1.14-1.67 | **0.0011** |
| *KLF15* | 231015_at | OS | 626 | 0.68 | 0.5-0.94 | **0.017** |
|  |  | RFS | 1764 | 0.66 | 0.56-0.77 | **1.50E-07** |
|  |  | DMFS | 664 | 0.67 | 0.48-0.92 | **0.014** |
